# Liposomal Form of the Echinochrome-Carrageenan Complex

**DOI:** 10.3390/md16090324

**Published:** 2018-09-10

**Authors:** Irina M. Yermak, Vladimir I. Gorbach, Valery P. Glazunov, Anna O. Kravchenko, Natalya P. Mishchenko, Evgeniya A. Pimenova, Viktoria N. Davydova

**Affiliations:** 1 G.B. Elyakov Pacific Institute of Bioorganic Chemistry, Far-Eastern Branch of the Russian Academy of Sciences, 100 Let Vladivostoku Prosp., 159, 690022 Vladivostok, Russia; vigorbach@bk.ru (V.I.G.); glazunov@piboc.dvo.ru (V.P.G.); kravchenko_25.89@mail.ru (A.O.K.); mischenkonp@mail.ru (N.P.M.); vikdavidova@yandex.ru (V.N.D.); 2 National Scientific Center of Marine Biology, Far-Eastern Branch of the Russian Academy of Sciences, Palchevskogo, 17, 690041 Vladivostok, Russia; eapimenova@yandex.ru

**Keywords:** carrageenan, echinochrome A, liposomes, mucine, mucoadhesive properties

## Abstract

Inclusion of drugs in liposomes offers the potential for localized and sustained delivery to mucosal surfaces. The inclusion of the carrageenan matrix with echinochrome A ((Ech)—the active substance of the drug Histochrome) in liposomes was studied. According to the spectral characteristics, Ech was not oxidized and retained stability after encapsulation in the liposomes and the lyophilization process. Loading the liposomes with negatively charged polysaccharide results in the increase in the zeta potential to more negative values (from −14.6 to −24.4 mV), that together with an increasing in the sizes of liposomes (from 125.6 ± 2.5 nm to 159.3 ± 5.8 nm) propose of the formation of the polymer coating on liposomes. The interactions of liposomes with porcine stomach mucin was determined by the DLS and SEM methods. The changes in the zeta-potential and size of the mucin particles were observed as the result of the interaction of liposomes with mucin. To evaluate the mucoadhesive properties of liposomes and the penetration of Ech in the mucosa, a fresh-frozen inner surface of the small intestine of a pig as a model of mucous tissue was used. Polysaccharide-coated liposomes exhibit very good mucoadhesive properties −50% of Ech remains on the mucosa.

## 1. Introduction

Marine organisms provide a vast source of various compounds with diverse biological properties and bioactivities. These biological properties introduce a wide range of new compounds that have pharmacological properties and make a significant contribution to the development of nanotechnology [1,2,3]. The development of multifunctional drug delivery systems has become a current and attractive concept in nanotechnology. Liposomal formulations were widely explored in the last decade for drug delivery application [4,5,6]. Liposomes are lipid vesicles that contain an aqueous solution of the active substance in their internal space or incorporate it into their shell. The function of liposomes strongly depends on their properties, and they are categorised based on their size, the number of phospholipid bilayers or the lipid charge [7,8]. The basic unit of the liposome consists of two key components, a hydrophilic head and a hydrophobic tail. The hydrophilic heads form the polar part (i.e., the central core) where hydrophilic pharmaceutical active ingredients are encapsulated, while the hydrophobic part, which consists of two fatty acid chains, can incorporate hydrophobic pharmaceutical ingredients [6,9]. Liposome-based delivery systems play an important role, owing to easy preparation and an increase in bioavailability, as well as offering drug targeting and controlled release. Liposomes have been investigated extensively for parenteral use but may also have potential in the local application of drugs in the eye [10] and in the oral cavity [11]. Liposomal forms of the antitumour drugs doxorubicin and vincristine [12] and the antifungal amphotericin B [13] are already successfully used in veterinary and human healthcare. The liposomal form of curcumin appears to be the best formulation for improving the bioavailability of curcumin in cells [5]. The long-term stability of liposomes can be immensely improved by coating them with various polymers [14,15]. Hydrophilic polymers are traditionally used as mucoadhesive materials in many formulations for transmucosal drug delivery [16]. Mucoadhesive polymers are flexible backbone macromolecules that contain hydrogen bonding groups that are capable of developing interactions with the glycoproteins present in the mucin. During contact with a mucous membrane, the polymers swell and thus expose the maximum number of adhesive sites, which enables inter diffusion and interpenetration of polymer chains and the mucin network. There are many polymeric materials that have been identified as mucoadhesive, e.g., poly (acrylic acid) PAA, poly (vinyl alcohol) PVA, chitosans, pectins, alginates, cellulose derivatives, hyaluronic acids and carrageenans [14,15,16,17,18,19,20,21,22,23]. Anionic polymers are the most widely employed mucoadhesive polymers within pharmaceutical formulation due to their high mucoadhesive functionality and low toxicity [14,16].

Anionic sulfated polysaccharides of red seaweeds—Carrageenans (CRG) exhibit a wide spectrum of biological activity such as antiviral, antimicrobial, anticoagulant, antitumor, immunomodulatory properties [3,24,25]. These polysaccharides have been used as a matrix for drug delivery in recent years [3,18] due to their diverse physicochemical properties and biological activities, which are used by pharmaceutical researchers to improve drug formulation and prolong drug release [25]. Carrageenans are linear galactans, whose basic structural units are disaccharide-carrabiose, consisting of alternating β-1,3- and α-1,4-linked galactose residues. Variations in the basic structure are determined by the content of 3,6-anhydrogalactose and by the location and number of sulfate groups [26]. CRGs had already been included in the US Pharmacopeia 35-National Formulary 30 S1 (USP35-NF30 S1), British Pharmacopeia 2012 (BP2012) and European Pharmacopeia 7.0 (EP7.0), with a promising future in the pharmaceutical industry. However, compared with commonly used mucoadhesive polymers, such as hydroxypropyl methylcellulose, chitosan, pectin and alginate, the utilization of CRG to coat liposomes has not been reported frequently. Recently, we showed that a water-insoluble sea urchin pigment, echinochrome (7-ethyl-2,3,5,7,8-pentahydroxy-1,4-naphthoquinone) (Ech), interacts with CRGs and is incorporated into the macromolecular structure of the polysaccharide [27]. Ech exhibits a wide range of pharmacological activities; it protects the mitochondrial functions of rat cardiac myoblast H9c2 cells from cardiotoxic agents and ROS initiators, such as doxorubicin, sodium nitroprusside and tert-butyl hydroperoxide, and it decreases the ROS level and mitochondrial membrane potential [28,29]. Ech with registration number P N002362/01 is the active substance in the cardioprotective and antioxidant drug Histochrome^®^, which is produced in Russia from the sand dollar [30] and is used for the treatment of ocular diseases, diabetic retinopathy, dystrophies, central retinal vein thrombosis, and post-traumatic haemorrhages [31]. The inclusion of Ech in complexes with CRGs decreased its oxidative degradation and improved its solubility [27]. This work is devoted to the inclusion in liposomes of the sulfated polysaccharides of CRGs that contain Ech. The use of CRG not only to include Ech in liposomes, but also for the possible coating of liposomes by polysaccharide can improve the stability of liposomes and increase mucoadhesive properties of the new drug form.

## 2. Results

### 2.1. Characterization of CRG and Ech

The carrageenan (CRG) was extracted from the algae *C. armatus* and was separated by using 4% KCl in a KCl-insoluble fraction. The structures of the KCl-insoluble polysaccharide were studied by 13C-NMR (Figure S1) and FT IR-spectroscopy (Figure S2), and the obtained spectra were compared with the spectra of polysaccharides that had been isolated earlier from these species of algae [32]. The identity of the spectra indicated that the KCl-insoluble fraction from *C. armatus* was κ-CRG (G4S-DA-carrabiose) with traces of more sulfated ℩-CRG (G4S-DA2S-carrabiose), which were randomly distributed along the polysaccharide chain as a single insertion according to our early data [33]. The viscosimetric molecular weight of CRGs, as calculated by the Mark-Houwink equation, was 560 kDa.

**Figure 1:**
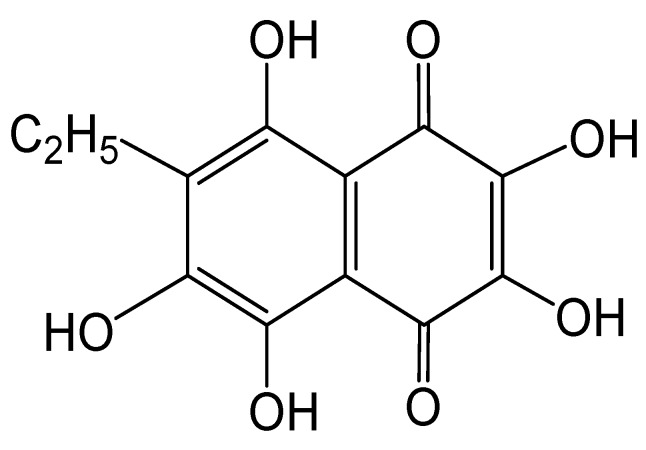
Structure of Ech.

The standardized substance echinochrome (pentahydroxyethyl-1,4-naphthoquinone, Ech) (Figure 1), was used as an ethanolic solution.

### 2.2. Preparation and Characterization of Conventional Liposomes

**Figure 2:**
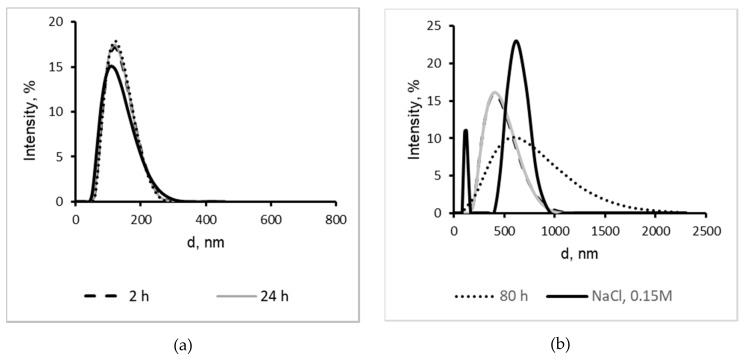
Hydrodynamic diameter distribution of (**a**) liposomes-100 and (**b**) liposomes-400 stored in apyrogenic water 2 h (black dash line), 24 h (grey line), 80 h (black dot line) and saline solution (black solid line).

Conventional liposomes were produced using standard thin film hydration and the sonication method. An extruder with membranes of 0.1 µm and 0.4 µm was used to reduce the heterogeneity of the liposomes. Unilamellar liposomal suspensions with a low polydispersity (pDI = 0.084 ± 0.015) were prepared with a membrane that had a pore size of 0.1 µm (liposomes-100). These liposomes were homogenic particles (Figure 2a) and had a monomodal distribution with a mean diameter of 125.6 ± 2.5 nm. Extrusion of conventional multilamellar liposomal suspensions using membranes with a pore size ≥0.4 µm did not produce unilamellar liposomes (liposomes-400). Liposomes produced with larger pore membranes yielded a more polydisperse suspension of particles (pDI = 0.135 ± 0.023) with a mean hydrodynamic diameter of 430.3 ± 29.8 nm (Figure 2b).

The stability of liposomes in solutions over a period of 2–24 h was studied with DLS. As seen in Figure 2, liposomes-100 are more stable than liposomes-400. They preserve their stability after 24 h storage in apyrogenic water or in a saline solution (Figure 2a). Aggregation and an increase in the polydispersity index (from 0.108 to 0.315) were observed after 24 h storage of the liposomes-400 in apyrogenic water (black dot line). The increase of the hydrodynamic diameter value from 430.3 ± 29.8 nm to 616.3 ± 45.7 nm was also registered.

### 2.3. The Entrapment Efficiency of Ech in Liposomes

Ech has very low solubility in water (<10^−5^ mole) and is soluble in saline solution with pH ≥ 7.0. We tried to incorporate Ech in phosphate buffer into liposomes, but in this case, the Ech oxidized rapidly and the obtained liposomes contained a non-native, but oxidized form of Ech. According to our earlier results, CRGs protected the Ech from its oxidative degradation and improved its solubility [27]. So it was not possible to incorporate the Ech without carrageenan into the liposomes. 

Water solutions of CRG/Ech were loaded into the liposome formulation using a standard thin film method followed by sonication. The butanol extraction was used to determine the concentration of Ech that was included in the liposomes, and the entrapment efficiency of Ech was determined by spectrophotometry at 468 nm. According to data, the entrapment efficiency of the Ech in the liposome was 48%. 

**Figure 3:**
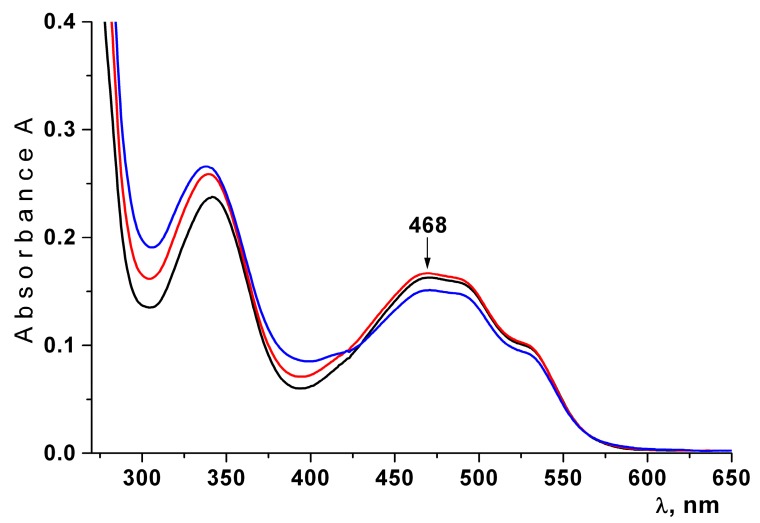
Absorption spectra of the Ech extracted with butanol from freshly prepared liposomes (black lines); from freshly prepared lyophilized dried liposomes (red line); from lyophilized dried liposomes after 30 days of storage at 4 °C (blue line).

The effect of lyophilization of the liposomes and their storage on the stability of Ech was studied. The stability of Ech in the liposomes was determined by measuring the absorption value at 468 nm. As shown in Figure 3, two characteristic absorption bands of the native Ech at 339 nm and 468 nm were observed in the absorption spectrum of Ech. It is known that the oxidazed form of Ech has an absorption band at 390 nm [34]. We did not observe such a band of absorption in the spectrum of Ech. According to Figure 3, the spectrum of Ech did not change after the lyophilization of liposomes and their storage. The obtained spectral characteristics indicate that Ech was not oxidized and was retained in liposomes in its native form.

With regards to the influence of lyophilisation on properties of liposomes (size distribution and zeta potential was insignificant), some increase in PDI value for liposomes-400 was observed (Table S1).

### 2.4. Characterization of CRG/Ech-Containing Liposomes

The particle sizes and zeta potentials of liposomes were measured immediately after sonication (Table 1). According to the electrophoretic mobility data, liposomes containing CRG/Ech had a negative charge. The ζ-potential value of liposomes CRG/EchA-100 and CRG/EchA-400 were −24.4 ± 2.7 and −5.6 ± 0.2, respectively. Inclusion of CRG/Ech into liposomes-100 led to a slight increase in their diameters, from 124.7 ± 2.2 to 159.3 ± 5.8 nm, and a decrease in the sizes of CRG/Ech liposomes-400 from 494.8 ± 4.0 to 419.5 ± 12.0 nm in comparison with that of the initial sample. The PDI of the liposomes was ≤0.23, which indicates the presence of a homogeneous liposomal population.

**Figure 4:**
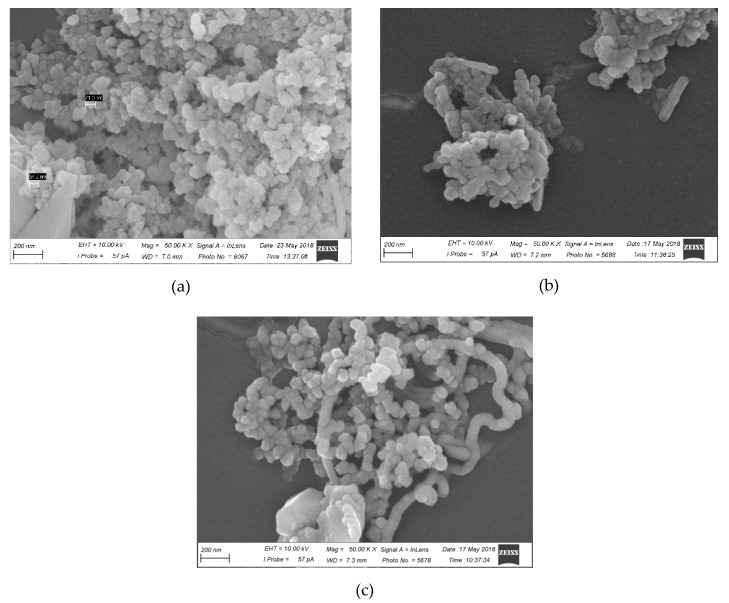

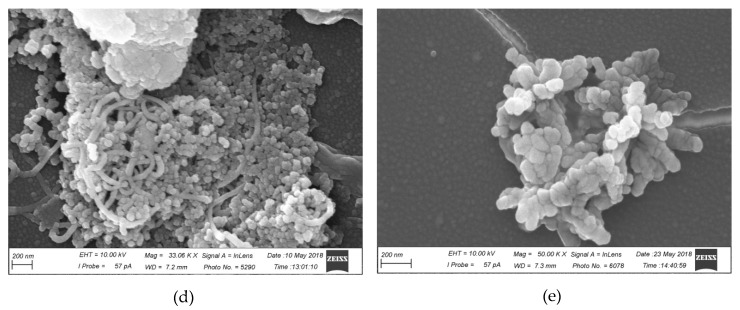
Scanning electron microscopy images of (**a**) CRG/ECH liposomes-100, (**b**) CRG/ECH liposomes-400, (**c**) mucin, (**d**) mucin-CRG/ECH liposomes-100 and (**e**) mucin-CRG/ECH liposomes-400. Mag. = 50.87 KX, EHT = 10.00 kV.

A scanning electron microscope (SEM) was used to study the microstructures of the CRG/Ech liposomes. SEM microphotographs of the produced liposomes are shown in Figure 4. SEM analysis revealed the formation of a spherical membrane of size 60–70 nm for liposomes-100 samples (Figure 4a). In addition, the images show a population of more heterogeneous vesicles for liposomes-400 (Figure 4b).

### 2.5. Interaction of Liposomes With Mucin

**Figure 5:**
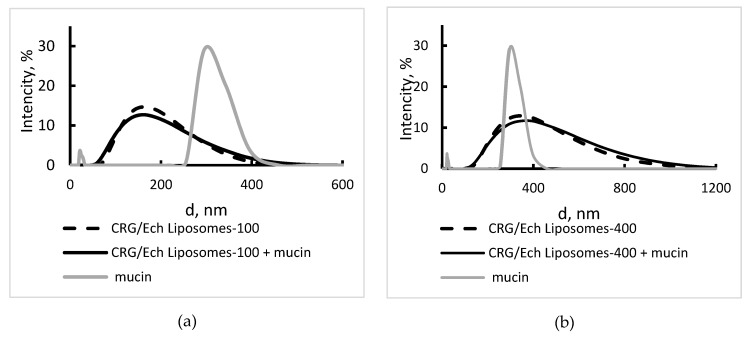
Hydrodynamic diameter distribution of (**a**) CRG/Ech liposomes-100 and (**b**) CRG/Ech liposomes-400 incubated in a mucin solution (0.15 mg/mL) for 2 h. CRG/Ech liposomes—black dash line, mucin—grey line, CRG/Ech liposomes with mucin—black solid line.

Mucin (0.15 mg/mL) in water is a polydisperse sample with a bimodal distribution (Table 1, Figure 5). The value of the Z-average was calculated according to the cumulant analysis (747 nm) and it indicated the presence a minor amount of large particles that were not recorded in the distribution analysis. The surface charge of mucin equaled −17.5 mV. The liposomes were incubated in a mucin solution (0.15 mg/mL) for 2 h and were analysed by DLS. The zeta potential of conventional liposomes in mucin solution was close to the value observed for pure mucin. Incubation of the CRG/Ech liposomes-100 in this solution led to an increased value of surface charge (from −24.4 to −20.8 mV), and the CRG/Ech liposomes-400 decreased the value of the zeta potential from −15.6 to −22.6 mV (Table 1). According to Table 1, in the CRG/Ech-containing liposomes, there were no noticeable variations in the hydrodynamic diameter (Figure 5) and the Z-average after storing the liposomes in the mucin solution.

A scanning electron microscope (SEM) was used to study the microstructures of the liposomes and their mixtures within the mucin. The mucin, as seen in the data presented in Figure 4c, forms a long thread-like structure in which the mucin vesicles are cross-linked into discrete networks. Liposomes-100 were built into the mucin mesh surface (Figure 4d), whereas liposomes-400 were collected in the aggregates (Figure 4e).

### 2.6. In Vitro Retention of Ech on Porcine Mucous Tissue

To evaluate the mucoadhesive properties of liposomes and the penetration of Ech on the mucosa, we used a fresh-frozen inner surface of the small intestine of the pig as a model of mucous tissue.

The loaded CRG/Ech liposome solutions were pipetted onto freshly excised porcine mucosa and were left in contact with the tissues. After 1 h, the content of the Ech in the liposome solution that was removed from the surface of the mucosa was measured using absorption at λ = 468 nm.

**Figure 6:**
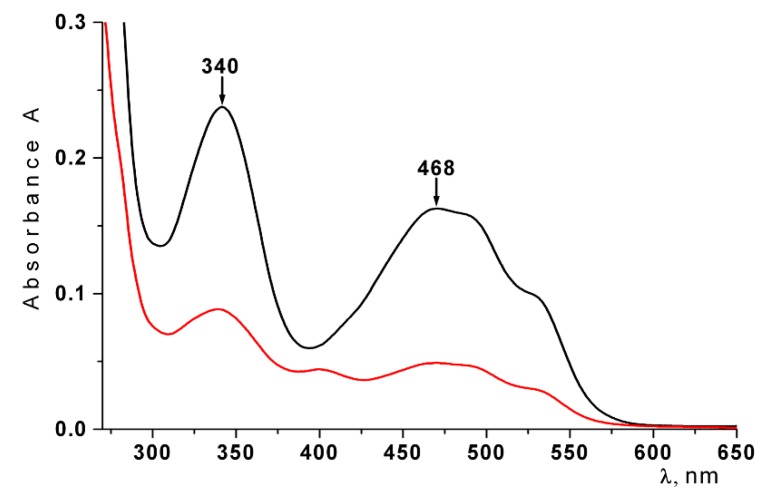
Absorption spectra of the Ech from freshly prepared liposomes (black line) and the liposome solution removed from the inner surface of the small intestine after an hour (red line).

As seen in Figure 6, a noticeable decrease in the absorption at 468 nm was observed in the spectrum liposome solution, which was removed from the inner surface of the intestinal mucosa. Calculations showed that approximately 50% of Ech remained on the mucosa.

## 3. Discussion

The red pigment of water-insoluble Ech from sea urchins is the active substance of the drug Histochrome and exhibits high antioxidant activity [30,31]. Recently, we have shown that the incorporation of Ech into the CRG matrix improves its water solubility and prevents Ech from oxidation [27]. In the present work, we included CRG/Eсh in the liposomes and examined the properties of these liposomes. Liposomes can encapsulate a wide range of drugs, both hydrophilic and hydrophobic, either in the aqueous core or in the lipid membrane. Many factors influence the encapsulation efficiency (% EE) and loading capacity of liposomes, including the drug/liposome ratio, the lipid composition, the presence of the charge, the method of preparation, and the chemical structure of the drug [35]. According to the data of Kandzija and Khutoryansky, water-soluble drugs have lower encapsulation in liposomes compared to their lipophilic counterparts [36]. Our data show that liposomes had the highest encapsulation efficiency related to Ech, which may be due to its hydrophobic nature and being stuck in the lipid bilayers, as was shown for the hydrophobic drug [10,24]. By coating the liposomes with a mucoadhesive polymer, the mucoadhesive properties and, thereby, the residence time on the mucosal surface, might be improved. Additionally, the coating can improve the stability of the liposomes [19,21]. In our work, CRG was used as a soluble matrix for the incorporation of Ech into liposomes and for the possible coverage of liposomes. According to the spectral characteristics, Ech was not oxidized and retained stability after encapsulation in the liposomes. The lyophilization process did not violate the native form of Ech. According to the data on DSL loading, the liposomes with negatively charged polymers of CRG resulted in the reversal of the zeta potential to negative values, which together with an increase in the sizes of liposomes-100 were proposed for the formation of the polymer coating. A less negative value was observed for the loaded liposomes-400. A higher value of the negative charge for liposomes-100, compared to that of liposomes-400, is probably due to a greater amount of CRG, which covers smaller liposomes. Loading of the liposomes with CRG resulted in a change in the hydrodynamic diameter. The liposomes-100 coated with CRG increased in size, while the liposomes-400 slightly decreased in size. SEM analysis reveals the formation of a spherical and small unilamellar membrane for coating liposome-100 and showed a population of homogenous vesicles. The PDI of CRG/Ech liposomes (≤0.23) is a measure of the size distribution and, according to the literature, the liposomal formulations are considered to be homogeneous if the PDI is ≤0.30 [37]. CRG has a high molecular weight (Mw = 5.1 × 10^5^) and may, therefore, form long tails and/or loops around the liposomes, which was shown for HM-pectin [19]. The surface properties of liposomes play an important role in their transport through the mucosa [38]. The epithelial cells of the mucosal tissues are coated by two types of mucins, membrane-bound and secreted (soluble) biomacromolecules that form a fully hydrated viscoelastic gel layer (mucus). The process of mucoadhesion involves a complex polymeric drug delivery platform with several types of binding mechanisms, such as electrostatic interactions, interactions with hydrophilic functional groups, or binding to the specific receptor sites in mucin [39,40].

The mucins, which are principal proteins that compose the dense fibre mesh of the mucus, are able to link particles by hydrophobic bonds (via globular inter-mucinic hydrophobic domains) and electrostatic interactions via negatively charged proteoglycans [16,39]. Mucoadhesive polymers are flexible backbone macromolecules that contain hydrogen bonding groups, which are capable of developing interactions with the glycoproteins that are present in the mucin. Chemically, the structure of CRG has many hydrogen-bond-forming groups, such as hydroxyl and sulfate groups, and this may allow for the interaction between CRG and mucin. By studying the interaction with mucin, the mucoadhesive properties of the drug delivery system can be estimated. The interactions of liposomes with porcine stomach mucin, which is similar to the mucin in the epithelium of the gastrointestinal tracts of humans, was determined by the DLS method. The changes in the zeta-potential of the mucin particle may be used as an indicator of the interaction of liposomes with mucin. The surface charge of the mucin particle was found to change upon the addition of liposomes. The Zeta-potential values of the mucin particles dropped from −17.5 to −20.8 mV or −22.6 mV in the presence of CRG-coated liposomes-100 and liposomes-400, respectively. An analysis of the surface charges of the CRG-loaded liposomes in the aqueous and mucin solutions showed different characteristics of interaction of the CRG/Ech liposomes-100 and the liposomes-Ech-400 with mucin. The ζ-potential of small liposomal Ech increases, approaches the value of pure mucin and decreases in the case of larger CRG/Ech liposomes-400. According to the results, it can be assumed that liposomes-100 can be incorporated into the pores of the mucin matrix without destroying it. The size of large particles with d ≈ 400 nm can exceed the pore size of the mucin network, and in this case, the destruction and interaction of liposomes with individual mucin molecules can be suggested. The data obtained by SEM are consistent with this assumption. Mucin can be adsorbed onto the CRG-coated liposomes-400.

Mucosal membranes are the moist surfaces that line the walls of various body cavities, such as the gastrointestinal tracts. They consist of connective tissues covered by an epithelial layer, the surface of which is covered by mucus [41]. Liposomes administered to various mucosal tissues are likely to be trapped by mucus via steric or adhesive forces, due to the mucoadhesive properties of the coated polymers [39]. During contact with the mucous membrane, the polymers swell and therefore expose the maximum number of adhesive sites, which enables the interdiffusion and interpenetration of polymer chains and the mucin network [16].

It was established that CRG-coated liposomes exhibited very good mucoadhesive properties. Our results showed that 50% of Ech remained on the mucosa even after washing with water. This can be due to the interaction between CRG and biological mucus, because mucoadhesive polymers may offer increased intimacy with the lining of the gastrointestinal tract and, hence, increase the bioavailability. It was shown that liposomes coated with pectin improved the drug delivery in the gastrointestinal tract [16]. Previously, we found that Ech A included in kappa-CRG has a moderate antiulcer effect. The activity of the CRG/Ech A complexes exceeded the activity of the Phosphalugel reference drug by three times the amount [27]. The liposome form of Escher carrus can enhance this effect. Moreover, the inclusion of Ech in liposomes covered with carrageenan opens up the possibility of its use as a cardiac buccal preparation.

## 4. Materials and Methods 

### 4.1. Algal Material

The representative species of red algae *Chondrus armatus* in sterile form was collected at Peter the Great Bay (Sea of Japan), which is near the border between the boreal and tropical zones: Order: Gigartinales, Family: Gigartinaceae—*Chondrus armatus* (Harv.) Okam. The algae were harvested at the end of August and were identified by Profs. Titlyanov E. and Titlyanova T. (National Scientific Center of Marine Biology, Vladivostok, Russia). The algae were washed with tap water in order to remove the excess salt. The seaweed was bleached by maintaining the specimens in pure acetone for 3 days prior to being air dried.

### 4.2. Extraction of Polysaccharide

The dried and milled algae (50 g) was suspended in hot water (1.5 L), and the polysaccharides were extracted at 90 °C for 2 h in a boiling water bath. The polysaccharides were separated into gelling KCl-insoluble and non-gelling KCl-soluble fractions, as described previously [32], and their structures were established according to a published protocol [32,33].

### 4.3. Molecular Weight Estimation

The viscosity average molecular weights of the carrageenan samples were calculated using the Mark-Houwink equation: [η] = KMα, where [η] is the intrinsic viscosity and K and α are empirical constants for κ-carrageenans, constituting 3 × 10^−3^ and 0.95 at 25 °C in 0.1 M NaCl, respectively, according to the literature data for this polymer-solvent system.

### 4.4. Fourier Transform-Infrared Spectroscopy (FT-IR)

IR spectra of the studied polysaccharides were recorded on 4000–600 cm^−1^ films on a Equinox 55 Fourier transform spectrophotometer (Bruker, Karlsruhe, Germany) with 4 cm^–1^ resolution. Sample preparation: The compound (8 mg) was dissolved in H_2_O (1 mL) and was heated at 37 °C on a polyethylene substrate until a dry film was produced. Then, the film was pressed between two NaCl plates and IR spectra were recorded. The spectra were normalized by the absorption of the monosaccharide ring skeleton at ~1074 cm^–1^ (A_1074_ ≈ 1.0).

### 4.5. Echinochrome

The standardized substance echinochrome (pentahydroxyethyl-1,4-naphthoquinone, Ech) (Figure 1), with a registration number in the Russian Federation of P N002362/01 (Russian State Register of Drugs (as of December 5, 2016) Part 2), was obtained from the G.B. Elyakov Pacific Institute of Bioorganic Chemistry, Vladivostok, in powder form. We used an ethanolic solution of Ech at a concentration of 10 mg/mL as a stock solution.

### 4.6. Preparation of Ech Water Solutions with Carrageenan

Ech was added to 0.5% carrageenan water solutions to a concentration of 0.1 mg mL^−1^. As a result, Ech water solutions with carrageenan at ratios of 1:5 (solution a) were obtained. The concentration of Ech A in the solution was measured using absorption spectra at λ = 468 nm.

### 4.7. Preparation of Liposomes

The liposomal formulations containing a fixed amount of egg lecithin and cholesterol were prepared using a thin film hydration method. At first, the solutions of the egg lecithin (10% in 1.6 mL) and cholesterol (69.9 mg × mL^−1^) were prepared in chloroform-methanol 9:1 (*v*/*v*) and mixed. The mixture was precipitated as a film on the wall of a glass test tube by evaporation of the solvent using a vacuum-line. The test tubes were then placed under a vacuum for at least 2 h to remove any residual solvent. Thin films of the lipid were hydrated and analysed.

### 4.8. Preparation of CRG/Ech-Containing Liposomes

A 1 mL solution of CRG/ Ech was added to the dried lipid film. The mixture was sonicated three times for 15 min in an ultrasonic generator, poured into 0.1 mL vials (1.5 mL), shaken and then centrifuged for 15 min at 15,000 *g*. The supernatant was removed and the pellet was suspended in 1 mL of water and again centrifuged. Washing was performed twice. Then, in each vial, 0.3 mL of water was added to the remaining precipitate to hydrate the liposomes. These liposome dispersions were sonicated in a bath for 30 min to reduce the size of the liposomes. An extruder was used to reduce the heterogeneity of the liposomes. The suspension of liposomes was passed through 0.1 and 0.4 micron membranes 10 times on an extruder according to the instructions. The resulting liposomes-100 and liposomes-400 were designated, respectively.

### 4.9. Measurement of Encapsulation Efficiency

Water (0.4 mL) and n-butanol (0.4 mL) were added to the suspension of liposomes (0.1 mL). The mixture was acidified with 20 μL of a 3 M HCl solution, shaken, incubated for 10 min in a US bath, and centrifuged for 20 min at 15,000 *g*. The upper butanol liquid layer was used to determine the Ech, and the aqueous layer was used to determine the content of carrageenan. The concentration of Ech in the solution was determined by spectrophotometry (Unicam 2 UV/VIS spectrophotometer) using absorption spectra at λ = 468 nm. The content of the encapsulated Ech (y, mg) was estimated from the difference between the total used drug (X) and the released drug. Thus, the encapsulation efficiency (EE) was calculated according to the equation, and a calibrated straight line was constrained in the same condition.

EE% = X/Y × 100%

The content of carrageenan-loaded liposomes was determined using a Taylor reagent (1,9-Dimethyl-methylene Blue, Sigma-Aldrich, Saint Louis, MO, USA). To this, 40 μL of aqueous extract was added to 200 μL of reagent. The mixture was incubated for 10 minutes, and the optical density was measured on a spectrophotometer (Bio-Tec Instruments, Inc., Auburn, CA, USA) at 535 nm.

### 4.10. Lyophilization of Liposomes

Sucrose (23.5 mg in each vial) as a cryoprotectant was added to the CRG/Ech-containing liposomes, prepared as described above. The mixture was sonicated in a bath, washed, and centrifuged, and the precipitate was suspended in water and dried by lyophilization for 12 h. The yield of freeze-dried liposomes was about 90%.

### 4.11. Preparation of the Liposomes with Mucin

A 1 mL mucin solution (0.15 mg × mL^−1^ or 5 mg × mL^−1^) in water was added to 1 mL of CRG/Ech-containing liposomes (concentration CRG = 0.5 mg × mL^−1^ and Ech 0.1 mg × mL^−1^) in water (pH 5.9), and then, it was stored for 1 or 18 h before analysis.

### 4.12. Dynamic Light Scattering (DLS) and Electrophoretic Properties of the CRG-Ech Complexes

The sizes of liposomes, their polydispersity index (PDI) and their ζ-potential values were determined using a ZetaSizer NanoZS system (Malvern, Cambridge, UK) operated at 633 nm. Each formulation was diluted 20-fold with ultrapure water. Prior to measurements, the samples were left for 1 h to allow the large aggregates to settle, because they can interfere with the measurements, even if their content does not exceed a low percent. The hydrodynamic diameters of the particles were automatically calculated using the software of the instrument based on an analysis of the autocorrelation function. The ζ-potentials were calculated from the experimentally determined electrophoretic mobility values using the Henry equation. Each sample was analysed three times at 25 °C, and the mean ± standard deviation were calculated.

### 4.13. Scanning Electron Microscopy Study

SEM images were generated using a Zeiss EVO 40 scanning electron microscope at an acceleration voltage 10.00 kV. Samples were prepared by pipetting a drop of liposomes suspension onto the surface of the Thermanox^®^ plastic coverslips (ThermoFisher Scientific, Fremont, CA, USA), fixed with 2.5% glutaraldehyde, incubated for 1 h for sedimentation, and dehydrated in alcohols of increasing concentrations and acetone.

Thereafter, the samples were completely dried in carbon dioxide according to the critical-point drying method by using a BALTEC 030 (BAL-TEC AG, Balzers, Liechtenstein). The samples were then placed on the surfaces of aluminium substrates and were coated with chromium.

### 4.14. In Vitro Retention Studies of Liposome on Porcine Intestine

The mucous tissue from the fresh-frozen inner surface of the small intestine of the pig was used as a model. Before the experiment, the mucosal tissue was thawed in the setting conditions, soaked in water and then placed in a solution of 0.1 M sodium chloride. Then, the mucosal tissue was fixed on a glass slide with the mucosal side facing upward, rinsed with 3 mL of NaCl solution and covered with liposomes (0.4 mL) to be tested. After an hour, the liposome solution was removed. The content of Ech in the solution was measured using absorption spectra at λ = 468 nm. Each experiment was conducted in triplicate

## Figures and Tables

**Table 1:** Sizes and ζ-potential of liposomes.

Sample	PDI	Z-Average, nm	Hydrodinamic Diameter		ζ-Potential, mV
			d, nm	Contents, %	
Conventional liposomes-100	0.084 ± 0.015	130.2 ± 4.4	125.6 ± 2.5	100	−14.6 ± 1.5
Conventional liposomes-400	0.135 ± 0.023	402.6 ± 12.4	430.3 ± 29.8	100	−8.2 ± 0.3
CRG/Ech Liposomes-100	0.132 ± 0.15	140.8 ± 1.0	159.3 ± 5.8	100	−24.4 ± 2.7
CRG/Ech Liposomes-400	0.219 ± 0.023	334.5 ± 1.4	419.5 ± 12.0	100	−15.6 ± 0.2
Mucin, 0.15 mg/mL	0.692 ± 0.070	732.2 ± 80.3	16.6 ± 1.2	6	−17.5 ± 1.6
			237.9 ± 14.0	94	
CRG/Ech Liposomes-100 + mucin	0.116 ± 0.014	140.0 ± 1.1	158.1 ± 3.3	100	−20.8 ± 2.9
CRG/Ech Liposomes-400 + mucin	0.227 ± 0.016	343.7 ± 2.3	408.1 ± 9.9	100	−22.6 ± 0.1

## Supplementary Materials

The following are available online at http://www.mdpi.com/1660-3397/16/9/324/s1. Figure S1. 13C-NMR spectrum of KCl-insoluble carrageenan from *C. armatus*; Figure S2. IR-spectrum of KCl-insoluble carrageenan from *C. armatus*. Bands 930 and 848 cm^−1^—κ-carrabiose; bands 930, 848 and 805 cm^−1^—℩-carrabiose; Table S1. Sizes and ζ-potential of liposomes before and after lyophilization.
