# Supplementary material for: Liposomal Form of the Echinochrome-Carrageenan Complex
Source: Mar Drugs. 2018 Sep 10;16(9):324. doi: 10.3390/md16090324 (PMC6163634; doi:10.3390/md16090324)
Supplement: Supplementary file 1 [file marinedrugs-16-00324-s001.pdf]

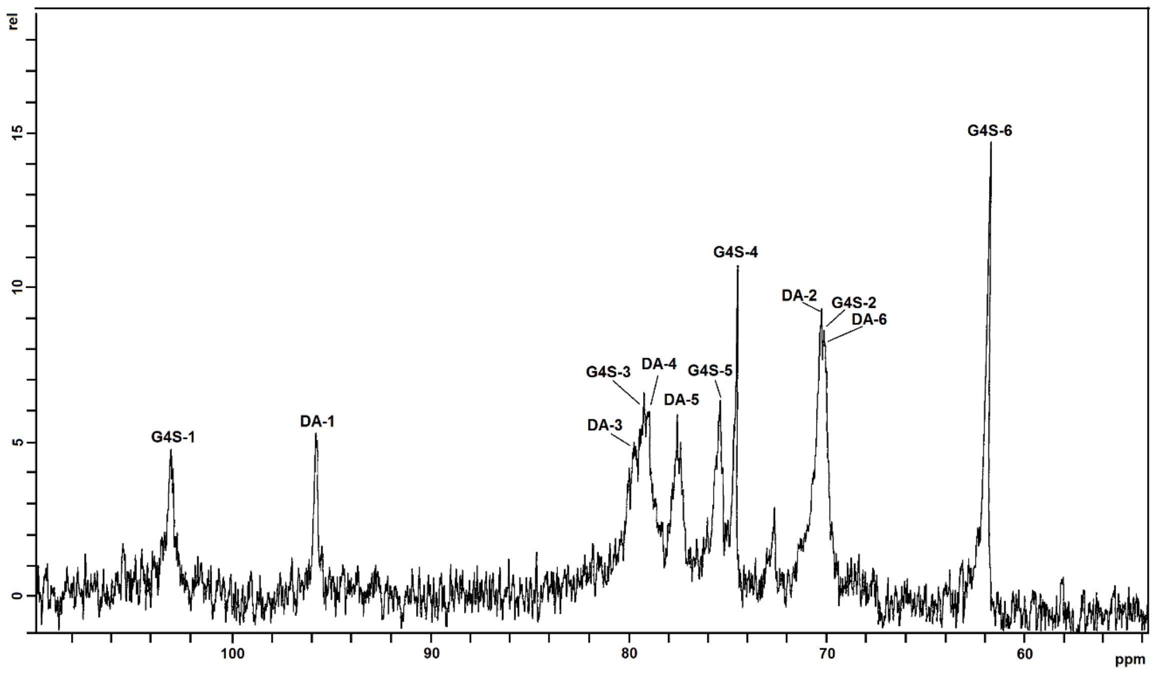

Figure S1. 13C-NMR spectrum of KCl-insoluble carrageenan from *C. armatus*

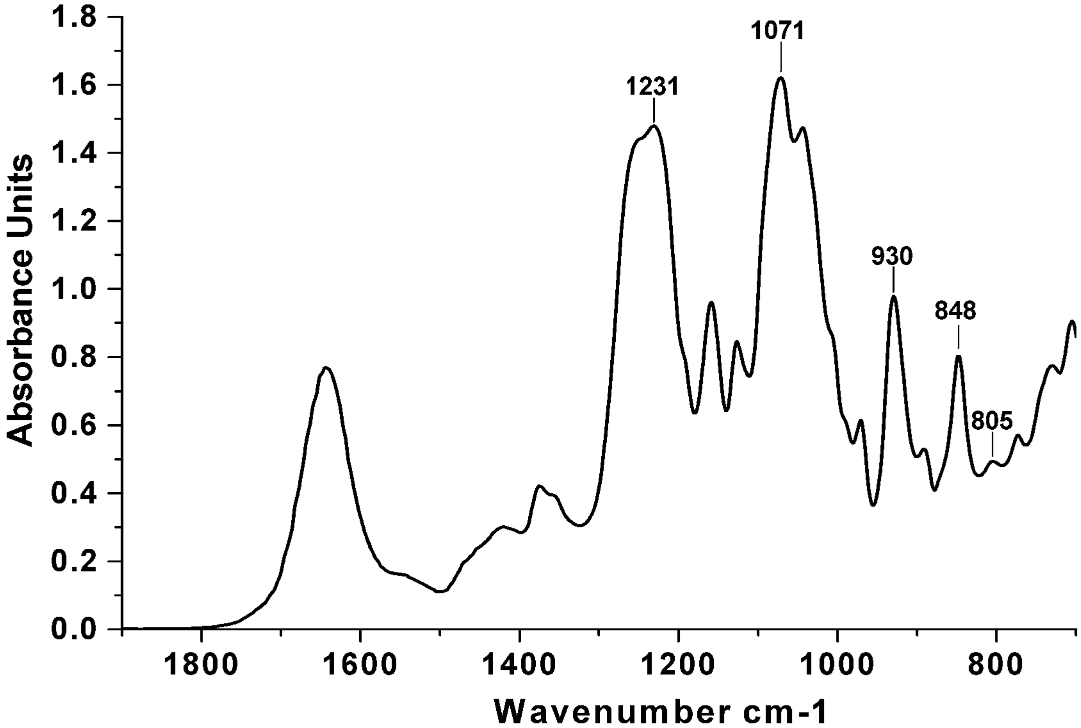

Figure S2. IR-spectrum of KCl-insoluble carrageenan from *C. armatus.* Bands 930 and 848 cm^{-1} – κ –carrabiose; bands 930, 848 and 805 cm^{-1} – ι-carrabiose

Table S1. Sizes and ζ-potential of liposomes before and after lyophilization.

Sample	PDI	Z-average, nm	Hydrodinamic diameter	ζ-potential, mV

			d, nm	Contents, %	
Liposomes-EchA-100	0.132±0.15	140.8±1.0	159.3±5.8	100	-24.4±2.7
Liposomes EchA -100 after lyophilization	0.305±0.005	167.2±1.9	153.7±10.1	100	-22.2±1.5
Liposomes-EchA-400	0.219±0.023	334.5±1.4	419.5±12.0	100	-15.6±0.2
Liposomes EchA -400 after lyophilization	0.251±0.008	308.0±2.3	370.3±11.3	100	-18.7±0.5
